# Semi-Supervised k-Star (SSS): A Machine Learning Method with a Novel Holo-Training Approach

**DOI:** 10.3390/e25010149

**Published:** 2023-01-11

**Authors:** Kokten Ulas Birant

**Affiliations:** Department of Computer Engineering, Dokuz Eylul University, Izmir 35390, Turkey; ulas@cs.deu.edu.tr

**Keywords:** machine learning, semi-supervised learning, classification, the k-star algorithm

## Abstract

As one of the entropy-based methods, the k-Star algorithm benefits from information theory in computing the distances between data instances during the classification task. k-Star is a machine learning method with a high classification performance and strong generalization ability. Nevertheless, as a standard supervised learning method, it performs learning only from labeled data. This paper proposes an improved method, called *Semi-Supervised k-Star* (SSS), which makes efficient predictions by considering unlabeled data in addition to labeled data. Moreover, it introduces a novel semi-supervised learning approach, called *holo-training*, against self-training. It has the advantage of enabling a powerful and robust model of data by combining multiple classifiers and using an entropy measure. The results of extensive experimental studies showed that the proposed holo-training approach outperformed the self-training approach on 13 out of the 18 datasets. Furthermore, the proposed SSS method achieved higher accuracy (95.25%) than the state-of-the-art semi-supervised methods (90.01%) on average. The significance of the experimental results was validated by using both the Binomial Sign test and the Friedman test.

## 1. Introduction

Entropy is a powerful model-building measure that has been used in the information-theoretic methods for data science domains. The k-Star method utilizes an entropic distance based on the information theory to measure the similarities among the data instances. It is one of the highly reliable and efficient machine learning (ML) methods [1]. Recently, it has been used in different kinds of fields, such as health [2], transportation [3], civil engineering [4], bioinformatics [5], agriculture [6], and mechanical engineering [7]. Despite the advantages of k-Star, a semi-supervised version of it has not been investigated yet.

*Semi-supervised learning* (SSL) is an ML technique focused on using labeled data as well as unlabeled data to perform a certain learning task [8,9,10]. It is especially practical and useful when a limited amount of labeled data exists. SSL is helpful since manual data labeling is a time-consuming, difficult, and expensive process. One of the most commonly used SSL approaches is *self-training*, which iteratively adds the predictions on unlabeled data into the initial labeled data for re-training the classifier. Nevertheless, self-training makes the final prediction based on a classifier alone, and using a single classifier may not be sufficient and efficient enough to provide a good separation between classes itself. Instead, one may build multiple classifiers to increase trustworthiness. Considering this motivation, we propose a novel SSL approach.

The proposed *holo-training* approach benefits from using three classifiers to increase predictive performance: (i) a *pre-classifier*, trained on the initial labeled data, (ii) a *pseudo-classifier*, trained on pseudo-labeled data, and (iii) a *post-classifier*, trained on the combined data. Our approach attempts to enrich the initial training data through the automatic labeling of some unlabeled data by combining multiple classifiers, resulting in improved prediction performance. Another important key point related to holo-training is that it is a direct method and not iterative like self-training.

The novelty and main contributions of this article can be listed as follows.

- It proposes an improved algorithm called *Semi-Supervised k-Star* (SSS).
- It introduces a novel semi-supervised learning approach called *holo-training*.
- The proposed holo-training approach outperformed the self-training approach on 13 out of the 18 datasets.
- Our method achieved higher performance (95.25%) than the state-of-the-art semi-supervised methods [11,12,13,14,15,16,17,18,19] (90.01%) tested on the same datasets.

The main motivation of this study was to improve the prediction accuracy of the models by incorporating unlabeled data into the learning process without requiring any human labeling efforts. Importantly, the aim of our study was to provide an algorithmic contribution towards increasing the classification accuracy of models compared to the previous studies. Through the experiments, the efficiency of the proposed SSS method was confirmed on 18 popular datasets. Different ratios of labeled data (10%, 25%, 50%, and 75%) were investigated for the k-Star algorithm. The significance of the experimental results was also validated by using both the Friedman test and the Binomial Sign test, considering a *p*-value < 0.05 as statistically significant.

The main organization of the paper is as follows. Section 2 presents various previous studies. Section 3 describes the proposed holo-training approach and the SSS method in detail. Section 4 provides the details of the experimental studies and obtained empirical results. Section 5 summarizes the main findings of the work and discusses possible future works.

## 2. Related Work

k-Star (also known as K*) [1] was developed as an effective learning method for both classification and regression problems. It is a typical example of lazy learning because it uses the existing training data when a query requests a response. The main difference between k-Star and other lazy learning methods is the use of the entropy concept for defining the distance metric instead of the classical Euclidean metric. Here, information theory helps in computing the distances among the data instances. 

In the literature, k-Star has been reported as the best-performing algorithm among other instance-based algorithms like k-nearest neighbors (KNN) and locally weighted learning (LWL) [7]. In many studies [2,3,4,5,6], it has been proven that k-Star performs better than other machine learning methods like the random forest, decision tree, neural network, and support vector machines.

The k-Star method has numerous advantages: (i) dealing with missing values [4], (ii) handling both symbolic and real-values attributes [7], (iii) addressing smoothness problems [20], (iv) being easy to implement, (v) dealing with imbalanced attributes and with noisy attributes [20], (vi) being regarded as a method for explainable artificial intelligence (XAI), (vii) combining attributes [20], and (viii) using both classification [2,3,7] and prediction [4]. Furthermore, since it is a lazy learning algorithm, the target function is locally approximated; thus, it can solve multiple problems simultaneously and deal effectively with changes in the application domain.

Based on the advantages of k-Star, this paper focuses on the research of a semi-supervised version of it. Unlike the traditional k-Star, its semi-supervised version benefits from unlabeled data and reduces the need for large amounts of labeled data in domains, thereby reducing the difficulty in labeling instances to increase the training set.

Semi-supervised learning is an ML technique in data science that focuses on utilizing both unlabeled and labeled data to perform a certain learning task [21,22]. Semi-supervised classification [23] has been usually studied in the field of SSL; however, some clustering tasks have also been performed [24]. Among the semi-supervised learning approaches, self-training is the most popular one. It is an iterative process, where at each step, a model is constructed to make predictions on unlabeled samples and select ones according to a threshold value. At the end of the cycle, a single final classifier is trained on the completely labeled set. However, self-training has several disadvantages: (i) The main disadvantage is that any initial mistakes made by the predictor or by the selection criteria can lead to assigning incorrect labels at each iteration. In other words, if a particular unlabeled data sample receives an incorrect label at any iteration, this error of the old model will propagate to the new models at the subsequent iterations. A classification mistake makes the learning performance worse and worse at each iteration. (ii) Another disadvantage of self-training is that an iterative solution increases the computational complexity compared to a direct solution, especially if the number of data instances is large. (iii) Self-training also has another drawback in that the careful selection of the threshold value is a difficult task. If the threshold is not selected appropriately, it can lead to erroneous predictions if noisy samples are classified as confident examples and added to the labeled training set. 

In order to overcome the limitations of self-training, we propose holo-training, which differs from self-training in several aspects. First, no threshold value is required in holo-training, and all available unlabeled data are assigned together with a class label. Thus, it allows us to perform semi-supervised learning without any parameter value selection and requires less domain knowledge. Second, holo-training is a direct method, while self-training is an iterative method. The direct solution is better than the iterative one in computational time and misclassification rates. Third, while self-training makes the final prediction based on a classifier alone, holo-training builds multiple classifiers to increase trustworthiness.

## 3. Proposed Method

One of the key points influencing the performance of classification methods is the amount of labeled data that is available in the training stage. It has been commonly accepted that the labeling process of a large volume of data is difficult, time-consuming, and expensive since it needs the involvement of human experts. In many application domains, unlabeled samples are easy to collect; therefore, they can be handled in a useful manner to improve the accuracy of classifiers. In this context, an improved algorithm, called *Semi-Supervised k-Star* (SSS), is proposed in this paper, aiming to effectively benefit from the large volume of unlabeled data as well as labeled data during the learning process. k-star is one of the classification algorithms whose semi-supervised performance has not been reported in the literature yet. 

The proposed SSS method is a lazy learning algorithm; that is, the class of a test data instance is predicted based on the class of the training examples similar to it. It differs from other semi-supervised lazy learners in that it utilizes an entropy-based distance function. 

The key property of the proposed SSS method is that it combines the predictions of multiple classifiers to assign correct labels to the unseen (new) data instances. Our method has the advantage of exploiting the diversity of the prediction power of the learned models by using different classifiers. The method is based on the *holo-training* approach, which is introduced in this paper for the first time and explained in Section 3.1.

**Figure 1:**
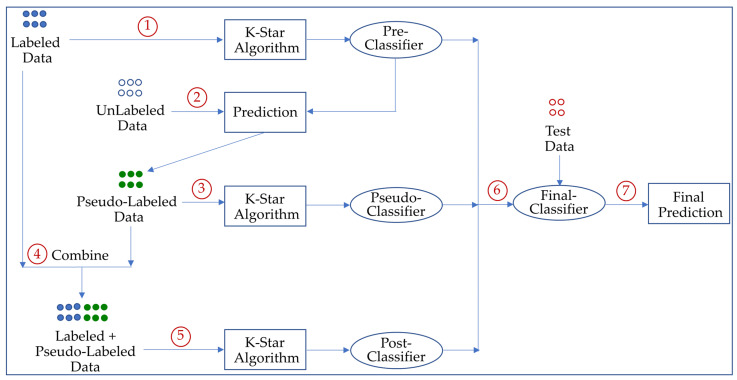
The general framework of the proposed SSS method.

The general overview of the proposed SSS method is illustrated in Figure 1. In the first step, a *pre-classifier* is initially trained with the original labeled data. In the second step, it was applied to the unlabeled data to generate pseudo-labels (i.e., labels that are assigned by a trained classifier instead of a human annotation). In the next step, a *pseudo-classifier* is then trained on the pseudo-labeled data. In the fourth step, the labeled and pseudo-labeled data are combined. After that, a *post-classifier* is built on the new augmented training set. In the training step, it uses the k-star algorithm in which the distances among the data instances are computed using information theory by utilizing an entropy-based function. In the test phase, three classifiers (pre-classifier, pseudo-classifier, and post-classifier) are applied to the given query instance for the classification task. Subsequently, the individual predictions of the three classifiers are combined via a majority voting scheme; namely, the final output is the one made by more than half of the classifiers.

### 3.1. Proposed Semi-Supervised Learning Approach: Holo-Training

*Semi-supervised learning* is one of the learning tasks that benefit from both unlabeled and labeled data instances. One of the common approaches in this type of learning is *self-training*, which iteratively increases the size of the training dataset by automatically assigning class labels to unlabeled samples with a threshold filter using a model trained in the previous iteration. It uses a single classifier in the final prediction phase. However, a single classifier alone may not be sufficient enough to capture the complex decision boundaries among classes and, thus, may not always be reliable for predictions. On the contrary, the use of multiple classifiers in a principled manner to improve discrimination performance is the main focus of our study. In other words, the main motivation behind our approach is that the methods that include multi-classifiers usually have higher accuracy than methods with one weak classifier. When we group multiple classifiers, with each one correcting the others’ misclassifications, we can construct one such strong model.

In this paper, we propose a new semi-supervised learning approach called *holo-training*. It benefits from using three classifiers to increase the prediction performance.

**Definition 1 (Pre-classifier).**  *A pre-classifier is a predictive model that is trained on the initial labeled data.*

**Definition 2 (Pseudo-classifier).**  *A pseudo-classifier is a predictive model that is trained on pseudo-labeled data.*

**Definition 3 (Post-classifier).**  *A post-classifier is a predictive model that is trained on the augmented data (labeled + pseudo-labeled data).*

To build a strong model, our approach employs knowledge of both labeled data and pseudo-labeled data, as well as augmented data. If the post-classifier is not good enough due to adding instances with incorrect class labels to the training set, this process may result in an increased probability of misclassification. For this reason, in our approach, the pre-classifier was also used in prediction since it was trained on human-labeled data, and thus it was probably a strong/robust classifier.

Clearly, the success of a semi-supervised approach is highly dependent on the newly labeled data based on its own predictions; thus, its weakness will probably lead to generating an incorrect training set and, therefore, building a poor classifier. In other words, adding mislabeled examples to the training set cannot provide much help to the learner. For this reason, the holo-training approach adds the unlabeled samples that are classified with the pre-classifier since this classifier has been trained on human-labeled data, and thus, it is probably a reliable enough classifier.

### 3.2. Formal Definitions

The k-Star algorithm benefits from information theory in computing the distances among the data instances. It uses an entropy-based distance measurement function to compute complexity by summing all possible transformations between two instances. This property provides a great contribution to its performance. Using an entropy-based distance function to measure the similarity makes it different from other lazy classification algorithms like KNN and LWL.

Let *I* be a set of instances and *T* be a finite set of predefined transformations on *I*. Let *t* be a value of the set *T* such that *t* ϵ *T*. Each *t* maps from one instance to one another, and thus it can be denoted as *t*: *I* → *I*. Here, *P* is a collection of prefix codes that is terminated by б (the stop symbol) to form T*. Each member of T* uniquely defines a transformation on *I* such that $\bar{t}$ = *t*_1_, *t*_2_, …, *t_s_*. In other words, a series of transformations is used to define the distance between two instances as the complexity of transforming one instance into another one. Let *p* be a probability function that is defined on T* and it has the following property: $\sum_{\bar{t}\epsilon R}p\left(\bar{t}\right)=1$. Now, P* is the probability function that is defined as the probability of all paths from instance *a* to instance *b*. The total probability is calculated as given in Equation (1). In other words, it represents each transformation from instance *a* to instance *b*.

(1) $$P^{*}\left(b|a\right)=\underset{\bar{t}\epsilon R:\;\bar{t\left(a\right)=b}}{\sum }p\left(\bar{t}\right)$$

K* is calculated by applying logarithm on P* in terms of complexity unit. The K* function is defined in Equation (2).

(2) $$K^{*}\left(b|a\right)=-\log_{2}P^{*}(b|a)$$

K* is a non-zero function such that K*(*b*|*a*) ≥ 0 and non-symmetric such that K*(*e*|*b*) + K*(*b*|*a*) ≥ K*(*e*|*a*).

By considering all possible transformations, the classification task is done based on the probability of transforming one instance into another. The probability of an instance *x* belonging to class *c* is found by summing the probabilities between each member of *c* and *x* as given in Equation (3).

(3) $$K^{*}\left(c|x\right)=-\underset{x_{j}\epsilon c}{\sum }\log_{2}P^{*}(x_{j}|x)$$

The probability of classifying a query instance for each class is calculated. The category with the highest probability is chosen as the class of the query instance.

Given a dataset D = { D_L_ U D_U_ } with n instances, where $D_{L}={\left\{\left(x_{i},y_{i}\right)\right\}}_{i=1}^{l}=\left\{\left(x_{1},y_{1}\right)\left(x_{2},y_{2}\right),\ldots ,\left(x_{l},y_{l}\right)\right\}$ represents labeled data and $D_{U}={\left\{x_{j}\right\}}_{j=l+1}^{l+u}=\left\{x_{l+1},x_{l+2},\ldots ,x_{l+u}\right\}$ denotes unlabeled data, l and u are the numbers of labeled and unlabeled instances, respectively, so n = l + u is the total number of instances. Here, the number of labeled instances is usually less than or equal to the number of unlabeled samples such as l <= u since labeled training data is scarce in many application domains, while unlabeled data is abundant, and manual labeling the instances is an expensive, time-consuming, and difficult process. Let $\left(x_{i},y_{i}\right)$ be an instance in the labeled dataset, $D_{L}$ denotes the i-th data point and consists of an m-dimensional sample $x_{i}$ ℝ from input space X such as x_i_ ϵ X and a corresponding label $y_{i}$, which is one of the k distinct classes such as $y_{i}\in Y=\left\{c_{1},c_{2},\ldots ,c_{k}\right\}$. Here, $y_{i}=\;c_{j}$ means sample x_i_ is from the j-th class in the predetermined label set. Since x_i_ forms from d-dimensional space, such as $x_{i}=\{x_{i}^{1},\;x_{i}^{2},\ldots ,x_{i}^{m}\}$, $x_{i}^{w}$ is the w-th attribute of the i-th data sample. We assume that Y has two or more labels and that both *D_L_* and *D_U_* are from the same data distribution. Let $X_{L}$ and $X_{U}$ be the sets of input spaces for the labeled and unlabeled instances, respectively, where $X_{L},X_{U}\in X$ and $X=X_{L}\cup X_{U}$. The objective of an SSL method is to obtain a robust learned hypothesis through *D_L_* U *D_U_* instead of *D_L_* alone.

**Definition 4 (Holo-training).**  *Holo-training is a new and efficient semi-supervised approach that builds three classifiers (pre-classifier, pseudo-classifier, and post-classifier) from labeled data (D_L_), pseudo-labeled data (D_P_), and augmented data (D_L_+D_P_), and aims to find a function f: X_L+P_ → Y that can accurately predict the class label y of a query data instance x.*

Given an unseen query instance *x* in the unlabeled data (*x* ∈ *D_U_*), the probability of *x* belonging to each class *c_i_* in the label set *Y* is calculated, and then the class with the maximum probability is selected as a candidate. Here, the goal is to select informative samples because the pseudo-labeled data with low probability may not improve accuracy; rather, it can decrease the performance due to misclassification. The formula for this process is given in Equation (4).

(4) $$H\left(x\right)=\underset{c_{i}\in Y}{\arg\max p\left(c_{i}|x\right)}\;\;\;\;\;\;where\;x\;\in \;D_{U}$$

where $H\left(x\right)$ is the maximum posterior probability of class for a given unlabeled data instance (*x*). 

The predictions of the three classifiers are considered together to make the final decision using a voting strategy. The formula of the majority voting strategy is given in Equation (5).

(5) $$\hat{Y}\left(x\right)={\arg\;\max}_{c\in Y}\overset{3}{\underset{t=1}{\sum }}d_{t,c}$$

where $d_{t,c}\in \left[0,1\right]$ is the decision of the *t*-th classifier, and $\hat{Y}\left(x\right)$ is the final output class that maximizes the equation for a given unseen input x. If the *t*-th classifier chooses class *c*, then $d_{t,c}=1$, and 0, otherwise. 

### 3.3. Proposed Algorithm: Semi-Supervised k-Star (SSS)

The basic structure of the SSS method is given in Algorithm 1. First, a *pre-classifier* is trained on the initial original labeled data *D_L_* with the k-Star algorithm. After that, the algorithm uses the pre-classifier for the prediction of an unlabeled dataset (*D_U_*) and to assign pseudo-labels to data points in this set. In the first loop, the newly assigned samples with their corresponding class labels are inserted into both the labeled training set (*D_L_*) and pseudo-labeled training set (*D_P_*) for the next learning cycle. The automatic pseudo-labeling loop terminates when all unlabeled samples have been assigned a label. In the next step, a *pseudo-classifier* is trained on pseudo-labeled data (*D_p_*), and a *post-classifier* is trained on the augmented data (*D_LP_* = *D_L_* + *D_P_*) using the k-Star method. Here, entropic distance measurement is used by each classifier. In the last loop, the query instances in the test set (*x* ∈ *TS*) are submitted to all classifiers (pre-classifier, pseudo-classifier, and post-classifier), and their estimations (*y*1, *y*2, and *y*3) are compared to each other. The SSS algorithm finds an agreement among the classifier predictions and assigns the final predicted label to the current data point at each iteration of the test process.
**Algorithm 1:** Semi-Supervised k-Star (SSS)**Inputs:***D_L_*: the labeled data with *l* instances such that *D* = {(*x_1_,y_1_*), (*x_2_,y_2_*),*….*, (*x_l_,y_l_*)}*D_U_*: the unlabeled data with *u* instances such that *D* = {*x_l+1_, x_l+2_,….*, *x_l+u_*}*TS:* test set to be predicted
**Output:***Ŷ*: Predicted class labels 
**Begin:***PreClassifier* = Train (*D_L_*)*D_P_* = Ø**foreach** (*x_j_*) **in** *D_U_**y_j_* = *PreClassifier* (*x_j_*)*D_L_*.Add (*x_j_*, *y_j_*)*D_P_*.Add (*x_j_*, *y_j_*)**end foreach***PseudoClassifier* = Train (*D_P_*) *D*_LP_ = *D*_L_ ∪ *D*_P_*PostClassifier* = Train (*D_LP_*)**foreach** *x* **in** *TS* **do***y*1 = *PreClassifier* (*x*)*y*2 = *PseudoClassifier* (*x*)*y*3 = *PostClassifier* (*x*)y = y1**if** (*y*1 = *y*2) **or** (*y*1 = *y*3)*y* = *y*1**else if** (*y*2 = *y*1) **or** (*y*2 = *y*3)*y* = *y*2**else if** (*y*3 = *y*1) **or** (*y*3 = *y*2)*y* = *y*3*Ŷ* = *Ŷ* ∪ *y***end foreach**
**End Algorithm**

The SSS method builds three models on the training sets *D_L_*, *D_P_*, and *D_LP_*. When the complexity of the base learner is stated as *T*, the training time complexity of the algorithm is given by O (T(*l*) + T (*u*) + T (*l* + *u*)), where *l* and *u* are the numbers of labeled and unlabeled samples, respectively.

### 3.4. Advantages of the Proposed SSS Method

The SSS method has a number of benefits as follows:

- The main advantage of SSS is the use of several classifiers jointly for better proficiency. It overcomes the limitation of the self-training approach by using the holo-training approach.
- In many real-life applications, a large amount of unlabeled data is available. Data labeling usually requires human efforts and it is a time-consuming process, especially when the number of class labels is large or the classes are similar to each other. The proposed SSS method deals with this problem by building a classifier to integrate unlabeled data with low-cost or no-cost.
- In this study, the holo-training approach was applied to the k-Star algorithm. However, it can be used with the integration of any other learner, such as a decision tree, support vector machine, or neural network. Since the method is entirely unaware of the base learner, with a small modification, it can simply use another classification algorithm to learn from both unlabeled and labeled samples.
- Another advantage of SSS is that it can be applied to any standard ML dataset without any prior knowledge about the dataset. In other words, SSS does not require any specific assumption for a given dataset.
- An important advantage is its implementation simplicity. After incorporating unlabeled data into the training set, the k-Star algorithm is applied easily. It also simply combines the individual predictions of multiple classifiers (pre-classifier, pseudo-classifier, and post-classifier) in a straightforward manner.
- Due to its simplicity and human-understandable properties, the proposed SSS method can be regarded as one of the XAI techniques at the intermediate level.
- The method inherits all the advantages from k-Star, such as dealing with missing values, handling both symbolic and real-values attributes, addressing smoothness problems, combining attributes, effectively dealing with changes in the training set, and using both classification and prediction.
- A huge amount of data collected in real-world applications is unlabeled. Since SSS covers a wide range of domains, it can enable numerous applications, and thus, it enlarges the application field of data science.

The limitation of the algorithm is that the space needed for the storage is larger as compared to some machine learning algorithms.

## 4. Experimental Studies

The experiments were conducted on 18 datasets to evaluate the efficiency and validity of the proposed SSS method. To show the superiority of the proposed *holo-training* approach, we compared it with the traditional *self-training* approach. We also investigated the impact of different ratios of labeled data (10%, 25%, 50%, and 75%) on the performance of SSS. Let *D* be the training data, consisting of a set of labeled data instances (*D_L_*) and unlabeled data instances (*D_U_*), and we define a labeled ratio as given in Equation (6).

(6) $$Labeled\;Ratio=\frac{\left|D_{L}\right|}{\left|D_{L}+D_{u}\right|}$$

where the |*D*| denotes the number of instances in dataset (*D*).

Our results were also compared with the results presented in the state-of-art studies [11,12,13,14,15,16,17,18,19] on the same datasets to show the superiority of the proposed SSS method. The proposed method was implemented in C# by utilizing the WEKA library [25]. The implementation of the method is available at https://github.com/KoktenUlasBirant/Semi-Supervised-k-Star (accessed on 4 January 2023).

For an enhanced analysis, all the datasets were partitioned according to the k-fold cross-validation technique. Each dataset was split into *k* unique groups of equal size. One of the groups was held out as a testing set, and the rest *k*-1 groups were utilized as a training set. The process was repeated *k* times, each with a different test set, and then the average accuracy of each repeated experiment was computed and reported. In this study, the parameter *k* was set equal to 10 since it has usually been selected by most studies in the literature. Since a random value was generated, each independent run obtained a different result. For this reason, each experiment was reported with an average of 5 independent runs with different random seeds. Briefly, the average accuracy over 5 iterations of 10-fold cross-validation for 4 different labeled data ratios (5 × 10 × 4 = 200) was reported as the performance result.

In our experiments, we used the accuracy measure to evaluate the performance of SSS. Accuracy is the proportion of correctly identified test samples to the total number of test samples. It is calculated according to the following formula:

(7) $$Accuracy=\;\frac{TP+TN}{TP+FP+TN+FN}$$

where true positive (*TP*) is the number of all correctly identified cases of the *c* class, false positive (*FP*) is the number of all cases outside the *c* class that are assigned to this class, true negative (*TN*) is the number of all cases outside the *c* class that are not assigned to this class, and false negative (*FN*) is the number of all misclassified cases of the *c* class.

To make the alternative methods comparable, we let them use the same parameter settings, and therefore, the only difference lay in the ability to manage unlabeled data. It should be noted here that in the self-training method, the threshold for the selection criteria was set to 0.75. This parameter was set to zero in the holo-training method since no threshold term was employed when developing it. The k-Star algorithm requires the setting of a single parameter, called the *global blending* parameter, which varies from 0% and 100%. It corresponds to the mixture value, which is related to entropy calculation. In this study, the default value (20%) was used for this parameter since it seemed to work well in previous studies [7]. 

### 4.1. Data Description 

To show the effectiveness of the SSS method, the experiments were performed on 18 widely used datasets taken from the UCI repository. These datasets have a different number of class labels, ranging from 2 to 24. Thus, we considered very different numbers of classes to obtain meaningful results regarding the performance of our method. While 11 out of them were binary class datasets, the rest were multi-class datasets. The features of most datasets comprise continuous or categorical values, while several datasets involve both of them. The dimensions of the datasets considered in this study varied from 3 to 70.

The brief descriptions of the datasets are summarized in Table 1. For each dataset, it presents the number of classes (#class), the number of instances (#instance), the number of features (#feature), and the types of the features (categoric, numeric, or both). As can be seen in the table, datasets encompassed diversity in different domains such as health, environment, automobile, and animal science. 

In the data preprocessing step, the ID columns in the datasets were removed because they had no effect on predicting the output. For example, the ID attribute was removed from the breast-cancer-wisconsin, labor, monks, parkinsons, and zoo datasets. Missing values were represented as unknown (“?”). All data files were converted to the Attribute-Relation File Format (ARFF) to provide standardization. The following data preprocessing steps were applied to the datasets:

▪ In the audiology dataset, the non-standard set of attributes was converted to a standard set of attributes according to the following rules. Each property was represented as a separate attribute. A property such as age_gt_60 was transformed to a Boolean attribute with values t and f. A property of the form x (y) was usually represented as a discrete attribute x whose possible values are the various y’s.
▪ In the parkinsons dataset, the class attribute was moved to the last position since the target (decision) attribute was set to the last index in the source code.
▪ In the labor dataset, collective attribute groups were combined into an overall view according to the ID values of instances since they were listed separately in the original data file. Furthermore, data related to positive and negative samples were brought together one after another.
▪ In the voting dataset and wine dataset, the first column was moved to the last index to make it the target class attribute.
▪ In the zoo dataset, each Boolean attribute was converted from 1 and 0 values to true and false values since these attributes indicate positive and negative conditions instead of numeric values. For example, the existence of the hair or tail of an animal was represented as a Boolean value according to whether the animal had it or not.

At each iteration of the k-fold cross-validation technique, each training set was split into two groups: labeled samples and unlabeled samples, since the aim of the study was to benefit from both of them. The datasets with thoroughly labeled training samples were used in this study. Hence, randomly selected samples were marked as labeled examples, and the labels of the rest were removed. In order to investigate the influence of the amount of labeled data, the training process was carried out by using a part of labeled samples. In the experiments, four ratios were used: 10%, 25%, 50%, and 75%. For instance, when the labeled rate was 10%, ten percent of data were randomly sampled as *D_L_* while the remaining 90% of the data were saved as *D_U_*. Although some of the samples were unlabeled, they were labeled during the training process by the proposed SSS method.

### 4.2. Experimental Results

We compared the proposed *holo-training* approach with the traditional *self-training* approach. In this experiment, we trained the classifier using both labeled and unlabeled samples. Here, we were not interested in the dataset itself; rather, we aimed to make explicit the differences in the learning processes between the two methods.

Table 2 shows the classification accuracy results achieved using the self-training and holo-training approaches for individual datasets. The first column in each sub-partition presents the accuracy obtained using the traditional self-training classifier, while the second column shows the classification results with our semi-supervised approach. The result of each method is the average accuracy obtained using 10-fold cross-validation over 5 runs. For the training process, labeled samples are selected randomly, and the resting ones are denoted as unlabeled samples.

The amount of labeled data that is available in the training stage is one of the major aspects affecting the performance of a classification algorithm. To understand the impact of the number of labeled samples on the performances of the methods, experiments were performed on the datasets at different scales (10%, 25%, 50%, and 75%) of labeled samples. For example, assume a dataset with 100 samples; when the labeled ratio is 10%, 10 instances were put into *D_L_* with their labels while the remaining 90 instances were put into *D_U_*. The sub-partitions in Table 2 show the proportion of labeled samples with respect to the cardinality of the dataset.

As can be observed in Table 2, holo-training achieved higher average accuracy than self-training when the scale of labeled data was taken as 10%. It is also observed that our approach achieved more satisfactory results with 25% labeled data. The same pattern is also valid for other scales (50% and 75%). It is clearly shown that SSS is an efficient semi-supervised method and can achieve high accuracy with fewer labeled samples. The prediction accuracy increases as the number of labeled data instances increases. For example, for the breast cancer dataset, increased accuracy values (91.74%, 92.85%, 94.45%, and 95.14%) were obtained as the labeled data size was increased. In short, the performance of the holo-training approach depends on the amount of labeled data since a large amount of labeled data can display the underlying structure of the data correctly, and therefore, the pre-classifier built on this data can also assign correct pseudo-labels to the unlabeled data.

As can be seen in Table 2, the results showed that the proposed holo-training approach usually achieved performance improvement over the self-training approach. For example, for the tic-tac-toe dataset, the proposed method outperformed its competitor in all four labeled ratios in terms of classification accuracy. This argument is also valid for the car-evaluation, flare, monks, Parkinson, and iris datasets.

According to the results, we can safely say that holo-training is superior to self-training with higher accuracy values. For example, when using only the 10% labeled part of the monks dataset, holo-training was able to accurately predict the correct labels of the unseen instances with an accuracy of 83.27%. On the other hand, self-training correctly classified the same dataset with an accuracy of only 80.98%. This is probably because of the fact that holo-training benefits from the collaboration of multiple classifiers, where an incorrect prediction of a classifier can be corrected by other classifiers, resulting in improved accuracy. This scheme is superior to the competing paradigm, which builds a single classifier from completely labeled and pseudo-labeled training datasets. Another reason for the performance improvement is that holo-training is a direct method instead of an iterative one, which can lead to propagating the error of an old model to new models at the subsequent iterations.

**Figure 2:**
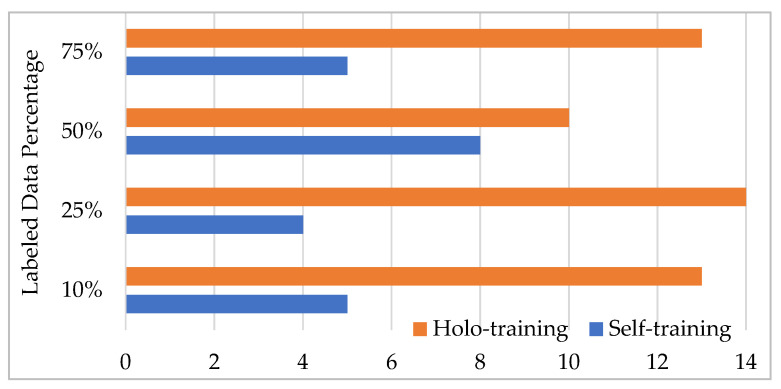
The number of wins of the semi-supervised learning approaches.

Figure 2 shows the number of wins when comparing the accuracies of the methods with respect to the labeled data ratio. It is observed from the figure that the proposed holo-training approach outperformed the traditional self-training approach regarding accuracy for most of the datasets. For example, it demonstrated a statistically significant difference for the ratio of 25% in 14 out of the 18 datasets. Similarly, holo-training achieved better performance than self-training in terms of accuracy in 13 out of 18 cases for ratios of 10% and 75%. In short, our method produced more wins on the datasets through all the experiments conducted.

**Figure 3:**
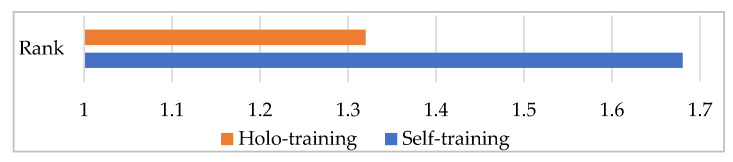
The rank of the methods under the accuracy metric.

Figure 3 visually shows the average ranks of the methods over all the datasets. This figure is useful for showing the difference between our method and the existing method under the accuracy metric. Specifically, we are interested in the success rank order of our method across a diversity of domains in which both unlabeled and labeled data are available. In the ranking technique, each method is rated with respect to its accuracy value on the corresponding dataset. This process is performed by giving rank 1 to the better method and rank 2 to the other one. In the case of a tie, the average ranking is assigned to each method. A lower rank indicates that the method has better performance. According to the results, our method achieved higher performance (1.32) on average, while the traditional method obtained an average rank of 1.68. Clearly, holo-training significantly outperformed its competitor on the accuracy metric.

Although the holo-training method has generally higher accuracy, we used both the Friedman test and the Binomial Sign test to compare our method with the existing method to determine whether there are significant differences between their performances. In other words, the non-parametric statistical tests were performed to investigate whether there was a pairwise performance difference between the algorithms. When computing the test statistics, accuracy values from two methods for the same datasets were considered paired observations. The null hypothesis (H0) was that the performances of two alternative methods were the same; otherwise, another hypothesis (H1) was present, which meant there were significant performance differences among the methods. The null hypothesis would be rejected if the *p*-value was less than a level of significance (*p*-value < 0.05). Based on the experimental results reported in Table 2, we computed the *p*-values of 0.00170 and 0.00109 using the Friedman test and the Binomial Sign test, respectively. Therefore, the results were statistically important because we obtained *p*-values that were smaller than the significance threshold (0.05). The results indicated that the null hypothesis was rejected with at least 95% confidence. This set of experiments demonstrated the strong performance of our method on the semi-supervised learning tasks.

In short, the experimental results showed that the SSS method possessed an improved accuracy in comparison to the existing semi-supervised methods. Moreover, using the holo-training approach, we obtained accuracies that were higher than the self-training approach. We obtained satisfactory results even when the number of labeled samples utilized was only a small fraction of the cardinality of the dataset.

### 4.3. Comparison with the State-of-the-Art Methods

The proposed SSS method was compared with the semi-supervised methods [11,12,13,14,15,16,17,18,19] previously applied to the same datasets. The comparative results are reported in Table 3 in terms of classification accuracies in percentage. Note that the results were directly taken from the corresponding original work since the researchers used the same datasets.

As can be observed from Table 3, our method obtained higher accuracy rates with the datasets than the existing methods. For example, when labeled data was 20% for the iris dataset, our method (94.27%) performed better than the VCNN+SVM [18] (90.66%) and LS-SVM [13] (93.33%) methods. Similarly, SSS (96.76%) demonstrated its superiority over the TWSVM [11] (94.44%) and SS-FMM [12] (94.60%) methods with the wine dataset. In short, SSS achieved an average accuracy of 95.25%, while other semi-supervised methods reached an average accuracy of 90.01%. Therefore, the proposed SSS method outperformed the others with a 5.24% improvement on average. As a result, our proposed method could be effectively used as a semi-supervised learning method to build a classification model by utilizing both labeled and unlabeled data in various domains.

## 5. Conclusions and Future Work

The k-Star algorithm is a convenient and effective supervised learning method that can be used for classification. It uses entropy as one of the information theory-based measures. This paper proposes its semi-supervised version (SSS), which makes efficient predictions by considering unlabeled data in addition to labeled data. It also introduces holo-training, which constructs a strong classifier by combining the information provided by three predictors using unlabeled and labeled samples in the training set simultaneously. In holo-training, the pre-classifier is built on the initial labeled data, the pseudo-classifier is trained on the newly labeled data, and the post-classifier is constructed on the augmented data (actual-labeled + pseudo-labeled). To test the ability of the proposed SSS method for classifying items belonging to the real-world domain, 18 popular datasets were used.

The main findings of the study can be listed as follows:

- It is the first study that used the k-Star algorithm for semi-supervised learning that achieved a high classification accuracy (>81%), hence proving to be efficient.
- Since it is a semi-supervised method, the SSS method efficiently increased the performance of the model while reducing the human labeling effort.
- The proposed holo-training approach achieved higher performance than the self-training approach in terms of average accuracy when the labeled data ratio was 10%, 25%, 50, and 75%.
- The accuracy rates slightly increase as the number of labeled data instances increases.
- In approximately 13 out of 18 datasets, holo-training outperformed self-training.
- Our method achieved a higher rank (1.3), while the traditional method obtained an average rank of 1.7.
- The proposed SSS method (95.25%) achieved higher accuracy than the existing semi-supervised methods (90.01%) on average.
- Our method demonstrated its superiority over the state-of-the-art methods [11,12,13,14,15,16,17,18,19] with an improvement of 5.24%.
- The Friedman test and Binomial Sign test results showed that self-training and holo-training have a statistically significant difference in their classification performance. The null hypothesis was rejected since the obtained *p*-values were 0.00170 and 0.00109, respectively.

In future work, an ensemble version of the SSS method can be implemented to benefit from the advantages of ensemble learning.

## Figures and Tables

**Table 1:** The descriptions of the datasets.

No	Dataset Name	#Class *	#Instance *	#Feature *	Categoric	Numeric
1	audiology	24	226	70	Y	N
2	blogger	2	100	5	Y	N
3	breast-cancer-wisconsin	2	699	10	N	Y
4	car-evaluation	4	1728	6	Y	N
5	colon	2	62	32	N	Y
6	flare	6	1389	12	Y	N
7	horse-colic	2	368	27	Y	Y
8	iris	3	150	4	N	Y
9	labor	2	57	16	Y	Y
10	led7digit	10	500	7	N	Y
11	monks	2	554	6	Y	N
12	parkinsons	2	197	22	N	Y
13	planning-relax	2	182	13	N	Y
14	tic-tac-toe	2	958	9	Y	N
15	titanic	2	2201	3	Y	N
16	voting	2	435	16	Y	N
17	wine	3	178	13	N	Y
18	zoo	7	101	17	Y	Y

* #Class: The number of classes, #Instance: The number of instances, #Feature: The number of features.

**Table 2:** Performance comparison of the proposed method (holo-training) with the existing method (self-training) in terms of accuracies over 18 datasets and 4 labeled ratios.

Dataset Name	10% Labeled		25% Labeled		50% Labeled		75% Labeled	
	Self-Training	Holo-Training	Self-Training	Holo-Training	Self-Training	Holo-Training	Self-Training	Holo-Training
audiology	41.92	43.59	54.63	56.44	65.49	66.20	73.28	72.91
blogger	67.20	67.60	73.20	73.60	79.00	79.00	83.60	83.40
breast-cancer-wisconsin	92.14	91.74	92.59	92.85	94.51	94.45	95.05	95.14
car-evaluation	70.18	74.25	70.03	75.60	73.06	78.14	81.33	82.29
colon	72.43	72.76	77.57	77.24	78.81	78.48	82.10	82.43
flare	61.93	69.40	67.14	70.87	70.60	71.82	72.02	72.64
horse-colic-surgical	74.40	74.27	76.93	77.14	79.00	78.53	80.60	80.67
iris	93.47	94.00	94.40	94.53	95.07	95.60	95.33	95.60
labor	76.00	77.47	83.40	82.73	89.20	88.53	90.80	91.87
led7digit	56.64	65.52	68.12	71.56	73.36	73.32	74.20	73.64
monks	80.98	83.27	87.59	90.83	93.76	95.88	96.64	97.08
parkinsons	78.29	78.92	81.24	81.95	86.19	86.21	89.06	89.17
planning-relax	58.96	58.83	57.67	57.45	58.04	57.92	59.54	60.41
tic-tac-toe	76.51	78.70	76.71	85.80	83.70	91.59	91.71	94.16
titanic	75.87	77.32	77.15	77.23	77.55	77.48	77.56	77.56
voting	91.16	91.12	90.89	91.63	91.99	92.32	92.41	92.32
wine	93.94	94.29	96.86	96.63	98.31	98.20	98.53	98.31
zoo	80.45	79.87	90.09	90.31	94.49	94.69	95.47	95.67
average	74.58	76.27	78.68	80.24	82.34	83.24	84.96	85.29

**Table 3:** Comparison of our method with the existing methods reported in terms of accuracy (percentage).

Reference	Labeled Data	Method	Dataset	Accuracy (%)	
				Previous Method	Proposed Method (SSS)
Goyal and Gupta [11]	30%	Twin support vector machine (TWSVM)	iris	86.67	94.40
			wine	94.44	96.76
Liu et al. [12]	30%	Semi-supervised fuzzy min-max neural network (SS-FMM)	iris	91.00	94.40
			wine	94.60	96.76
Jiang et al. [13]	20%	Least square support vector machine (LS-SVM)	iris	93.33	94.27
Forestier and Wemmert [14]	16%	Semi-supervised learning enhanced by multiple clusterings (SLEMC)	iris	93.01	93.87
	13.5%		wine	90.05	94.72
Liu et al. [15]	Avg 10%–90%	Semi-supervised kernel extreme learning machine (SK-ELM)	iris	84.00	95.02
	Max 10%–90%			95.00	95.60
Zhao et al. [16]	10%	Sparse congruency representation (SCR)	iris	85.00	94.00
Liu et al. [17]	50%	Semi-supervised mixed label propagation (SMLP)	iris	82.00	95.60
			wine	94.00	98.20
Han et al. [18]	20%	Vector centroid neural network (VCNN) + Support vector machine (SVM)	iris	90.66	94.27
			wine	91.33	96.86
Nandedkar and Biswas [19]	10%	Reflex fuzzy min-max neural network (RFMN)	iris	85.00	94.00
			average	90.01	95.25

## Data Availability

The datasets are publicly available at the following website: https://archive.ics.uci.edu/mL/datasets.php (accessed on 12 December 2022).
